# Suicide by self-immolation in southern Iran: an epidemiological study

**DOI:** 10.1186/s12889-020-09778-z

**Published:** 2020-11-03

**Authors:** Ali Akbar Mohammadi, Mohammadreza Karoobi, Amirhossein Erfani, Reza Shahriarirad, Keivan Ranjbar, Mitra Zardosht, Mahboobeh-Sadat Modarresi, Zari Afrasiabi

**Affiliations:** 1 Burn and Wound Healing Research Center, Plastic and Reconstructive Surgery Ward, Shiraz University of Medical Sciences, Shiraz, Iran; 2 Thoracic and Vascular Surgery Research Center, Shiraz University of Medical Sciences, Shiraz, Iran; 3 Student Research Committee, Shiraz University of Medical Sciences, Shiraz, Iran

**Keywords:** Suicide, Burn, Self-immolation, Prevalence

## Abstract

**Background:**

Self-immolation, as a method of suicide, is one of the most violent and extreme ways which is usually attempted by the ignition of inflammable materials, with more than 70% fatality rate. In the literature, Iran has been reported to have a high rate of self-immolation; therefore, this study aimed to evaluate the prevalence and epidemiological features of self-immolated patients.

**Methods:**

In this retrospective cross-sectional multicenter study, data from burn patients from 2007 till 2017 due to self-immolation and suicide were enrolled in our study.

**Results:**

Based on our data, 657 out of 3530 burn patients (18.6%) with a mean age of 31.15 (SD = 0.452) were documented as suicidal attempts; the majority were female (63.2%) and married (66.3%). Most of the patients were from rural areas (58.3%) with an education level of under diploma (63.2%). Of the patients in our study, 22 (8.7%) had comorbid systemic diseases and 115 (50.5%) had psychiatric disorders.

**Conclusion:**

Due to the high prevalence of suicide by self-immolation among the Iranian population, further studies to evaluate the risk factors and clarify the high-risk group for more targeted approaches are recommended.

## Background

Suicide is considered as a public health burden in most countries and accounts for 800,000 deaths annually in the world [1]. Furthermore, it is considered as the third reason for death among individuals ranging from 15 to 44 years all over the world and in the United States as the sixth cause of mortality among the youth (15–24 years) [2]. A report by the World Health Organization (WHO), stated that the majority of suicides occur in middle and low-income countries [3].

Suicide methods vary depending on the cultural characteristics of the community, including hanging, poisoning, alcohol or drug overdose, self-immolation, gunshot, suffocation, jumping, exsanguination, and gas (carbon monoxide) inhalation [4, 5]. Self-immolation as a way of suicide is one of the most violent and extreme ways which is usually attempted by the ignition of inflammable materials, such as gasoline (petrol) or kerosene (paraffin) with more than 70% fatality rate [6–10].

The deliberate action of self-immolation is mostly reported in mid and low-income countries in Asia and Africa and happens rarely in developed ones. Additionally, the risk factors for self-immolation varies; most victims in developed countries tend to be older men, while in low and middle-income countries it tends to be among younger women. Moreover, the most frequent predisposing factor in self-immolation in Iranian and non-western population has been reported to be adjustment disorders, while psychoses, addictions, and major depression were reported among western countries [11].

In literature, Iran has been reported to have a high rate of self-immolation with some regions reaching almost 22.4 cases per 100.000 individuals every year [12–15]. Further studies in Iran demonstrated that the self-immolation rate is higher in younger women compared to others with a rate of 4.5 cases in every 100,000 population that comprise 16% of all burnt hospital admissions. Most of the studies conducted in Iran reported mental health issues as the most important risk factor for self-immolation. Moreover, higher prevalence of self-immolation among Iranian women has also been reported in previous studies [16–18].

Based on these results, we aimed to determine the prevalence of self-immolation and suicide in adults who referred to Ghotbeddin Shirazi and Amir al-Momenin burn hospitals in Shiraz and to explore the causes and potential risk factors to provide data for designing a preventive program for those who are at risk in near future.

## Methods

This cross-sectional study was carried out from 2007 to 2017 in Amir-al Momenin and Ghotbeddin Shirazi Burn Hospital, which are tertiary referral burns and plastic surgery healthcare centers affiliated to Shiraz University of Medical Sciences, Shiraz, Iran. The subjects consisted of burn victims who were registered as outpatients and in patient subjects. Each patient’s information was reviewed and their demographic characteristics were obtained from the burn registry including age, sex, marital status, residence, burn etiology, burn percentage, and suicide motivation. The confidentiality of the patients’ information was secured by removing the names and any personal information aside from the previously mentioned data collection. The database registry was initiated by Burn and Wound Healing Research Center located in Amir-al Momenin Hospital. Elements for this database questionnaire were designed by clinicians and epidemiologists. All the statistical analyses were performed by the statistical package for social sciences (SPSS Inc., Chicago, Illinois, USA) version 26.0. Data are presented as mean ± SD and proportions as appropriate and frequency of the groups were presented as valid percentage. The relationship between the categorical variables was evaluated with specific chi-square tests (X^2^).

## Results

During this study, based on the data registry and out of a total of 3530 patients, 657 (18.6%) were documented as suicidal attempts. The patients’ age ranged from 16 to 86 years (mean = 31.15, SD = 0.452). The majority of the patients were female (63.2%), married (66.3%), and between the ages of 16 to 45 years (87.1%). Most of the patients were from rural areas (58.3%) with an education level of under diploma (63.2%). Among the patients in our study, 22 (8.7%) had comorbid systemic diseases and 115 (50.5%) had psychiatric disorders. Table 1 demonstrates the demographic features of patients in our study.

**Table 1:** Demographic features, along with the severity and causative agents of self-immolation among adults referring to Shiraz Burn hospitals during 2007–2017

	Self-immolation *n = 657*		Total *n = 3530*		P.value
	Frequency	Percentage (%)	Frequency	Percentage (%)	
**Sex**					< 0.001
Male	242	36.8	2148	60.8	
Female	415	63.2	1382	39.2	
**Age (years)**					< 0.001
16 to 45	572	87.1	2679	75.9	
45 and above	85	12.9	851	24.1	
**Marital status**					0.034
Single	203	31.3	985	28.3	
Married	430	66.3	2439	70.1	
**Residence**					< 0.001
Urban	274	41.7	1660	47	
Rural	383	58.3	1869	53	
**Education**					< 0.001
Illiterate	110	16.9	684	19.5	
Under Diploma	412	63.2	1906	54.3	
Diploma	94	14.4	568	16.2	
Student	5	0.8	303	8.6	
University Degree	31	4.8	49	1.4	
**Location**					< 0.001
Kitchen	24	3.9	496	14.4	
Bath	23	3.7	99	2.9	
Room	198	31.8	953	2.9	
Yard	219	35.2	575	16.6	
Workplace	3	0.5	525	15.2	
Tent	2	0.3	32	0.9	
Outdoors	153	24.6	642	18.6	
**Comorbid diseases**					0.733
Epilepsy	6	27.3	54	21.3	
Renal disease	2	9.1	10	3.9	
Diabetes	8	36.4	54	21.3	
Anemia	1	4.5	3	1.2	
Asthma	1	4.5	8	3.1	
Hypertension	1	4.5	18	7.1	
Thyroid disease	1	4.5	13	5.1	
Migraine	1	4.5	10	3.9	
Hermaphrodite	1	4.5	1	0.4	
**Source**					< 0.001
Fire	610	95.8	1752	51.8	
Explosive	26	4.1	1130	33.4	
Electrical	1	0.2	228	6.7	
**Percentage of burn**					< 0.001
< 20%	40	6.1	1071	30.3	
20–40%	120	18.3	1110	31.5	
40–60%	131	19.9	584	764	
> 60%	366	55.7	765	21.6	

Statistical analyses demonstrated a significant correlation among age, sex, education, marital status, location of occurrence, and living place with self-immolation among the population. (*P* < 0.05). There was also a significant correlation in the source of injury and percentage of burn with self-immolation among the population. (*P* < 0.001).

Based on our results, the source of self-immolation among the majority of patients was fire (95.8%) with the burn percentage ranging from 3 to 100% (mean 63.69, SD = 1.07). Table 2 demonstrates the features regarding the cause and severity of self-immolation among patients in our study.

**Table 2:** Motivation of suicide among self-immolation patients referring to Shiraz hospitals during 2007–2017

	Frequency *n = 657*	Percentage (%)
**Motivation**		
Unemployment	2	0.3
Familial Issues	500	76.1
Financial	11	1.7
Mental or Psychiatric disorder	121	18.4
Death Of Beloved	7	1.1
Guilt	15	2.3
Chronic disease	1	0.2

## Discussion

Suicide is a significant public health problem not only in Iran, but also in other western countries [1–3]. Moreover, self-immolation as a suicidal act is very uncommon in developed countries; nevertheless, its rate in developing countries is increasing [6–8]. Reports indicated that Iran had a high prevalence of self-immolation among most countries [12]. Self-immolation accounts for almost 16% (7.5 to 36.6%) of all hospitalizations due to burn injuries [19]. Furthermore, with an average range of 22.4 cases per 100,000 population, many regions of Iran have the highest rate of self-immolation worldwide [13–15].

Regarding the rates of suicide attempts in southern Iran, reports demonstrated an annual rate of 4758 to 4857 cases [20, 21]. Based on our results, 657 cases of self-immolation were reported in 10 years, demonstrating an annual rate of 65.7 cases. Therefore, we conclude that 1.4% of all suicide attempts in our region are due to self-immolation, which is supported by other studies [20, 21].

In this study, the mean age of the individuals who committed self-immolation was 31 and the majority of patients were in the age range of 16 to 45 years. Studies in other countries reported the same results; the mean age was reported to be 31.2 years in Durban, South Africa [22], and 27 years in Eastern Sri Lanka. Other research conducted in developed countries pointed out similar or higher mean age in their studies, i.e. 30 years in Queensland, Australia [23], and 38 in Verona, Italy; Ontario, Canada [24–26]. It seems that self-immolation in developing countries is mostly seen in adolescents, whereas it tends to be more frequent in older individuals in developed countries. This can be explained by the fact that Iran’s population is divided into various age groups with a higher proportion of younger adults in comparison to western countries [27]. Nevertheless, unemployment among the youth, especially for the educated population, can be considered as a cause of dissatisfaction and frustration which would lead to self-burning suicide [13], while age patterns of self-immolation among the American population indicate that a significant percentage of victims might have been influenced by mental and/or substance-related diseases [28].

Studies on suicide demonstrate that suicide leading to death is more common among men, while among women attempting suicide has been recorded more frequently [29]. In our study, a large proportion of patients were female (63.2%) and married (66.3%). Studies in Russia and Italy have shown no sex differences among their patients [24], whereas the majority of immolators in developed countries were males; as to marital status, other studies indicated the same result, as predominate immolators were married [27, 30, 31]. It seems that young married women with a lower level of education are the main victims of suicide via self-immolation; from a socio-cultural perspective, the reason that causes women to be more susceptible to inflict self-burning injuries at an early age compared to men is the probability of more exposure to social and familial stress, much earlier than men. This can be explained by the fact that in developing countries like Iran, women tend to get married earlier than men. Therefore, we believe that family therapy and marriage counseling can play an important role in preventing self-immolation.

In our study, the majority of patients lived in rural areas (58.3%) with an education level of under diploma (63.2%). Other studies indicated that most of the immolators were illiterate people or had a low level of education [30, 32]. A report by Ahmadi in Kermanshah demonstrated that 53% of their cases which committed suicide via self-immolation were living in rural areas [4]. The key element of the social setting for self-immolation in rural areas might be the cultural background. Studies specifically analyzing this subject described several relevant cultural factors like marriage and divorce-related cultural practices regarding self-immolation; for instance, the cultural sense of self-immolation in rural parts of Iran is to interpret it as a path for women with family problems to emancipate themselves, but the main issue eventually comes back to the lack of mental health care in these areas [33].

Different situations have been reported to motivate an individual to perform self-immolation, including disputes between family members ranging from verbal conflicts to physical abuse, especially in partners, parents, or children, as demonstrated in our study (76%) and other similar studies [4]. Other risk factors include legal or occupational issues; chronic illness or death of a loved one; lower socioeconomic status (economic deprivation, unemployed); low educational levels; restricted access to healthcare, in particular for psychological health and counseling services; and lack of spiritual beliefs or moral principles [4, 34–38].

As mentioned in the results of our study and other studies, the majority of patients had a low level of education [13, 15, 31, 34, 35]. A potential reason for this is that education significantly improves the individual’s temperament and increases one’s understanding of circumstances of everyday life and social dynamics and empowers them to be able to better voice their grievances about any injustices and easily gain their rights; this leads to discouragement of self-defeating actions and indulgence in suicide behaviors.

Fire was the most commonly used tool for self-burning in our study (95.8%). Researches indicated that the most common material used as a fire accelerant was kerosene or gasoline [35]. This is close to the reports from other provinces of Iran, India, and other parts of the Middle East, while in Europe, and North America, gasoline was the frequently used tool [36]. Other studies have mentioned in interviews with patients who survived self-immolation that easy access to flammable liquids such as kerosene from thermal devices was the key factor affecting their decision in choosing the method of suicide. While various factors play a part in this action, easy access cannot justify this act, but can be avoided by limiting it [37].

The approach to primary prevention of self-immolation can be divided into two strategies. The first one is the strategies targeting high-risk individuals and those aiming the whole population. Throughout the “high-risk approach”, those who are considered at high risk of developing a certain disease are identified and interventions are coordinated by provider-based measures to reduce their disease difficulties. These interventions are essential in health care, but their expense per person can be high [39]. The suicide prevention researches indicate that counseling and therapies are relatively successful in minimizing the likelihood of suicide [38]. This requires the use of professional therapy services in local regions to be reinforced and institutionalized. These initiatives may include a community understanding of the negative impacts of excessive life stress. Religious leaders may play a vital role in inspiring and mobilizing the religious population to grow against this threat by engaging in preventive services and supporting therapies as a traditionally accepted method of coping with mental illness symptoms and preventing self-harming impulsive actions.

The second strategy or population strategy is directed at modifying disease-related actions, environmental influences, and their social and economic corollaries in society as a whole, and a high proportion of the community will benefit from much less costly modifications than the “high-risk approach”. The population strategy is considered to be a cost-effective approach regarding the reduction of disease rates [39]. Policies affecting on larger scales including increasing prospects for employment and reducing poverty may limit the portion of the population at risk of experiencing mental illnesses and traumatic aspects of life associated with self-immolation also the role of the mainstream media (radio, television) and high school and college curricula in helping to alter public perception around the mental disease, disability, and rehabilitation require further research.

There were some limitations in our research; firstly, this study was conducted on hospitalized patients due to unavailability of all records of self-immolation data in the whole province. The second limitation includes the inadequacy of psychological analysis on patients due to incoordination of hospital staff with patients’ families.

## Conclusion

This research demonstrates that self-immolation in many areas of Iranian society is a dynamic trend and a significant health issue should be considered as a mental health problem in our society. Therefore, implantation of strategists and programs to prevent and decrease suicide rates is essential. Further studies in this filed which evaluate the risk factors and clarify high-risk groups for more targeted approaches are recommended.

## Data Availability

SPSS data of the participant can be requested from the authors. Please write to the corresponding author if you are interested in such data.

## References

[1] World Health Organization. Global Health Estimates 2013: Deaths by Cause, Age and Sex, Estimates for 2000–2012. Geneva: WHO; 2014. http://www.who.int/healthinfo/global_burden_disease/en. Accessed 19 Mar 2020.

[2] Suhrabi Z, Delpisheh A, Taghinejad HJIjob, trauma (2012). Tragedy of women's self-immolation in Iran and developing communities: a review. Int J Burns Trauma.

[3] World Health Organization. Preventing suicide: a global imperative. Geneva: WHO; 2014. https://www.who.int/mental_health/suicide.prevention/world_report_2014/en. Accessed 19 Mar 2020.

[4] Ahmadi AJJobc, research (2007). Suicide by self-immolation: comprehensive overview, experiences and suggestions. J Burn Care Res.

[5] Rezaeian MJAjodm (2008). Epidemiology of suicide after natural disasters: a review on the literature and a methodological framework for future studies. Am J Disaster Med.

[6] Mohammadi AA, Danesh N, Sabet B, Amini M, Jalaeian HJJobc, research (2008). Self-inflicted burn injuries in Southwest Iran. J Burn Care Res.

[7] Peck MDJB (2012). Epidemiology of burns throughout the world. Part II: intentional burns in adults. Burns..

[8] Laloe V, Ganesan MJB (2002). Self-immolation a common suicidal behaviour in eastern Sri Lanka. Burns..

[9] Pham TN, King JR, Palmieri TL, Greenhalgh DGJTJobc, rehabilitation (2003). Predisposing factors for self-inflicted burns. J Burn Care Rehabil.

[10] Laloë VJB (2004). Patterns of deliberate self-burning in various parts of the world: a review. Burns..

[11] Ahmadi A, Mohammadi R, Schwebel DC, Khazaie H, Yeganeh N, Almasi AJB (2009). Demographic risk factors of self-immolation: a case–control study. Burns..

[12] Parvareh M, Hajizadeh M, Rezaei S, Nouri B, Moradi G, Nasab NEJB (2018). Epidemiology and socio-demographic risk factors of self-immolation: a systematic review and meta-analysis. Burns..

[13] Ahmadi A, Mohammadi R, Stavrinos D, Almasi A, Schwebel DCJJobc, research (2008). Self-immolation in Iran. J Burn Care Res.

[14] Saadat M, Bahaoddini A, Mohabatkar H, Noemani KJB (2004). High incidence of suicide by burning in masjid-i-Sulaiman (southwest of Iran), a polluted area with natural sour gas leakage. Burns..

[15] Ahmadi A, Mohammadi R, Schwebel DC, Khazaie H, Yeganeh N, Almasi A (2010). Demographic risk factors of self-immolation. Inj Prev.

[16] Maghsoudi H, Garadagi A, Jafary GA, Azarmir G, Aali N, Karimian B (2004). Women victims of self-inflicted burns in Tabriz, Iran. Burns.

[17] Mohammadi AA, Danesh N, Sabet B, Jalaeian H, Mohammadi MJIjoms (2008). Self-burning: a common and tragic way of suicide in Fars Province, Iran. Iran J Med Sci.

[18] Fardiazar Z, Sadeghi-Bazargani H, Mohammadi RJIjogm (2012). Domestic injuries and suicide among women of reproductive age in Iran. Int J Gen Med.

[19] Groohi B, Alaghehbandan R, Lari AR (2002). Analysis of 1089 burn patients in province of Kurdistan, Iran. Burns..

[20] Naghshvarian M, Kaveh MH, Hesampour M, Rezaee F, Mirahmadizadeh AR (2016). Epidemiologic study of suicidal attempt cases in Fars Province, south of Iran, 2010-2011. J Health Sci Surveill Syst.

[21] Mirahmadizadeh A, Rezaei F, Mokhtari AM, Gholamzadeh S, Baseri A (2020). Epidemiology of suicide attempts and deaths: a population-based study in Fars, Iran (2011–16). J Public Health.

[22] Sukhai A, Harris C, Moorad RGR, Dada MA (2002). Suicide by self-immolation in Durban, South Africa. Am J Forensic Med Pathol.

[23] Cameron DR, Pegg SP, Muller M (1997). Self-inflicted burns. Burns..

[24] Castellani G, Beghini D, Barisoni D, Marigo M (1995). Suicide attempted by burning: a 10-year study of self-immolation deaths. Burns..

[25] Ho WS, Ying SY (2001). Suicidal burns in Hong Kong Chinese. Burns..

[26] Shkrum MJ, Johnston KA (1992). Fire and suicide: a three-year study of self-immolation deaths. J Forensic Sci.

[27] Zarghami M, Khalilian A (2002). Deliberate self-burning in Mazandaran, Iran. Burns.

[28] Thombs BD, Bresnick MG, Magyar-Russell G (2007). Who attempts suicide by burning? An analysis of age patterns of mortality by self-inflicted burning in the United States. Gen Hosp Psychiatry.

[29] Sarkhel S (2009). Kaplan and Sadock's synopsis of psychiatry: behavioral sciences/clinical psychiatry, 10 [sup] th edition. Indian J Psychiatry.

[30] Dibii A, Gharebayhi R (2000). Study of suicidal burns in Ahwaz. Iran J Legal Med Org IR I.

[31] Ahmadi A (2005). Frequency of self-immolation in attempted suicide patients in west Islam Abad city (1997–2003). J Behbood.

[32] Rastegar AL, Alaghehbandan R (2003). Epidemiological study of self-inflicted burns in Tehran, Iran. J Burn Care Res.

[33] Khankeh HR, Hosseini SA, Rezaie L, Shakeri J, Schwebel DC (2015). A model to explain suicide by self-immolation among Iranian women: a grounded theory study. Burns..

[34] Ahmadi A (2007). Suicide by self-immolation: comprehensive overview, experiences and suggestions. J Burn Care Res.

[35] Ahmadi M, Ranjbaran H, Azadbakht M, Gorji MH, Gorji AH (2014). A survey of characteristics of self-immolation in the northern Iran. Ann Med Health Sci Res.

[36] Ghaleiha A, Khazaee M, Afzali S, Matinnia N, Karimi B (2009). An annual survey of successful suicide incidence in Hamadan, Western Iran. J Res Health Sci.

[37] Rezaie L, Hosseini SA, Rassafiani M, Najafi F, Shakeri J, Khankeh HR (2014). Why self-immolation? A qualitative exploration of the motives for attempting suicide by self-immolation. Burns..

[38] Ahmedani BK, Vannoy S (2014). National pathways for suicide prevention and health services research. Am J Prev Med.

[39] Amnesty International (2009). Demand dignity: dying too young: maternal mortality claims the life of one woman every minute.

